# Supplementary material for: Mineral dust increases the habitability of terrestrial planets but confounds biomarker detection
Source: Nat Commun. 2020 Jun 9;11:2731. doi: 10.1038/s41467-020-16543-8 (PMC7283277; doi:10.1038/s41467-020-16543-8)
Supplement: Supplementary file 1 — Supplementary Information [file 41467_2020_16543_MOESM1_ESM.pdf]

Supplementary information for

Mineral dust increases the habitability of terrestrial planets but confounds biomarker detection

by Boutle et al.

Supplementary Note 1

Dust distribution and feedback mechanisms

Supplementary Figure 1 shows the vertically integrated distributions of atmospheric dust for one of the small and one of the large categories (or size bins). Suppl. Fig. 1a and b demonstrate why dust has such a strong effect on a tidally-locked planet. The primary dust uplift region is the sub-stellar point on the day-side, where the near surface winds are very strong, forced by inflow to the convecting region. These strong surface winds lift large amounts of dust into the atmosphere. Whilst the larger sizes quickly return to the surface, the smaller sizes are lifted to the height of the super-rotating jet, which provides an incredibly efficient mechanism of mixing the dust throughout the planetary atmosphere.

The quantitative results therefore depend on how much dust in the small categories can be lifted from the surface, which contains two key uncertainties. Firstly, how much surface dust in these categories exists. On unvegetated regions of Earth, and Mars, dust of this size is abundant, and therefore it does not seem unreasonable to assume it would be produced on any similar rocky planet. Secondly, the tuning of the dust parametrization, which links well understood uplift from point-sources to grid-box mean uplift in the general circulation model. Our model is tuned to replicate this process well on Earth. To investigate these uncertainties, Supplementary Figure 2 shows the surface temperature from two additional simulations, for comparison to Figure 3. In Suppl. Fig. 2a, the multiplicative factor (k_1), used to scale the grid-box mean friction velocity into a point-source friction velocity applicable for use in the uplift equation[1], is reduced to 2 from its control value of 2.2. This change represents a large, but not unrealistic, variation in this parameter based on recent model tuning exercises, and due to the nonlinearity of uplift actually represents a $\approx 30\%$ reduction in uplift. In Suppl. Fig. 2b, this change is combined with a $\approx 50\%$ increase in dust in the lowest 3 size-bins (and commensurate reductions in the upper 3 bins), thus allowing increased uplift of the most radiatively important dust sizes. As shown, both modifications lead to small quantitative changes to the results presented, but the qualitative difference from the NoDust simulation remains unchanged.

Suppl. Fig. 1c and d show why the mechanism does not act quite as efficiently on a non-tidally-locked planet, and consequently why the effect of dust is always to cool these planets. Surface wind-speeds are much weaker in the equatorial convecting region, reducing the dust uplift. Therefore, the total amount of dust within the atmosphere is lower for the nTL case. However, the effect on the outer-edge of the habitable zone is controlled more by the resultant atmospheric dynamics. The mid-latitude storm track winds provide most of the poleward transport of dust, but this mechanism is nowhere near as effective at transporting dust to the coldest regions of the nTL planet as the super-rotating jet is for the TL case. Therefore the polar dust concentrations are very low indeed, and so whilst they will still produce a similar long-wave forcing or heating as dust produces on the night-side of the TL case, the magnitude of this heating is much reduced. The polar temperature is therefore dominated by heat transported from equatorial regions, which with dust present is much colder due to short-wave scattering increasing the albedo and reducing the temperature of equatorial regions (Suppl. Fig. 3a – d).

Therefore, the coldest temperature of any planet with an Earth-like atmosphere is controlled by two competing mechanisms; transport of warmer air from regions strongly heated by stellar radiation, and long-wave emission from dust particles transported to these regions. On a tidally-locked planet, the increase

in temperature due to long-wave emission from the dust is stronger than the decrease in direct heat-transport from the day-side, therefore the night-side warms. On a non-tidally-locked planet, the decrease in heat-transport from equatorial regions is stronger than the increase due to long-wave emission from dust, therefore the polar regions cool. Hence the contrasting effects on the outer-edge of the habitable zone.

Whether these mechanisms hold for thick CO_2 dominated atmospheres is a matter for future studies, although we speculate that they should. The dominant factor remains the ability to transport dust to the coldest regions of the planet, which should be qualitatively unaffected provided the TL planet retains an equatorial super-rotating jet.

Supplementary Figure 3 provides further detail on the inward movement of the inner-edge of the habitable zone. In particular, Suppl. Fig. 3e and f show the large reduction in stratospheric water vapour caused by dust, via the increased short-wave reflection and consequent reduction in surface temperature. Although demonstrated here for the non-tidally-locked planet, the mechanism works identically on a tidally-locked planet.

Supplementary Note 2

Sensitivity of dust to land surface configuration

Supplementary Figure 4 explores the differences between simulations adopting the land-surface configurations shown in Figure 2. As discussed in Lewis et al.[2], land positioned on the day-side of a tidally-locked planet is the most interesting and useful case to consider, because any land located on the night-side is likely to be locked away under permanent ice-caps and so be unable to influence the carbon-silicate cycle or be available for dust uplift. As shown (Suppl. Fig. 4a and b), the dust has a much greater impact, both warming the night-side and cooling the day-side, when the land is arranged into a small, contiguous land mass rather than a large, disparate archipelago.

A fuller analysis of the effect of the introduction of a continent can be found in Lewis et al.[2], but one of the key consequences for us is the emergence of desert regions on the continental land mass. The dry land surface in these regions (near the east and west edge of the continent) allows for significant uplift of dust (Suppl. Fig. 4c and e). A direct consequence is a significant cooling (> 25 K) of these desert regions due to the scattering effect of the dust, a cooling that actually provides a slight negative feedback on the dust effect, lowering the temperature of some of the desert regions below freezing and thus locking dust into the surface, preventing further uplift. Similar to the 100% land planet shown in Fig. 3, the smaller dust sizes are efficiently mixed throughout the atmosphere (Suppl. Fig. 4c), contributing significantly to the long-wave radiation budget of the night-side of the planet, giving rise to substantial warming (Fig. 2a and Suppl. Fig. 4a). The extreme change shown in the stratospheric water-vapour (Fig. 2b) arises because this is a planet which can still sustain a strong hydrological cycle in the NoDust scenario, and therefore stratospheric water-vapour contents are similar to aquaplanet experiments[3]. The extreme cooling induced by the dust substantially reduces the strength of the hydrological cycle and thus limits the export of moisture into the stratosphere. The dust reduces the stratospheric water-vapour content down to amounts comparable to the 100% land simulations, which are very low indeed.

By contrast, the experiment in which each grid-cell contains land and ocean retains a circulation very similar to the aquaplanet simulations. Therefore the strong hydrological cycle results in a land surface which is uniformly wet, suppressing uplift of dust from the surface. Atmospheric dust contents (Suppl. Fig. 4d and f) are therefore much lower than the continental arrangement, explaining the more muted effect on surface temperatures and stratospheric water vapour contents (Fig. 2 and Suppl. Fig. 4b). Its important to note that even in this case, the dust is still providing a sizeable perturbation to the climate system, in particular the reduction in stratospheric water-vapour content.

In summary, the key difference between TL land surface configurations shown in Fig. 2 is due to the emergence of deserts in continental configurations, which unsurprisingly produce a large source of dust which can have planetary scale feedbacks.

Supplementary Note 3

TRAPPIST-1 observables

To understand how the observability of dust or potential biomarker gases could vary depending on the distance and apparent magnitude of the parent star, Supplementary Figure 5 presents the same results shown in Figure 4 but with the star moved further away, such that its apparent magnitude is similar to TRAPPIST-1 rather than Proxima Centauri. These simulated observations are therefore indicative of potential observations of TRAPPIST-1e.

As shown, for nearer (brighter) stars (Fig. 4), the separation of a dusty from a non-dusty transmission spectrum is clearly identifiable in the synthetic observations with relatively few transits (i.e. an amount that might be observationally feasible with orbital periods of tens of days). Similarly, the peaks of the potential biomarker gases are broadly identifiable. The effect of dust is to absorb or scatter stellar radiation passing through the planetary atmosphere across all wavelengths, which in turn makes the planet obscure a much larger portion of the stellar disk. However, as the star is moved further away (becomes dimmer), the number of transits required to separate the spectra increases, such that with JWST it is unlikely that the presence of dust could be observed for TRAPPIST-1 with a reasonable number of transits; it would require hundreds of transits to clearly identify a difference in the spectra. This is also true of the biomarker gases, the peaks of which are now much reduced for the dimmer star (Suppl. Fig. 5c) and therefore much harder to identify with a small number of transits. These results are broadly consistent with those of Fauchez et al.[4], who also showed that it was only likely to be the CO_2 peak which could be identified with $10 - 20$ transits, with many more required for any other feature. However, future instrumentation may improve this.

Supplementary Figures

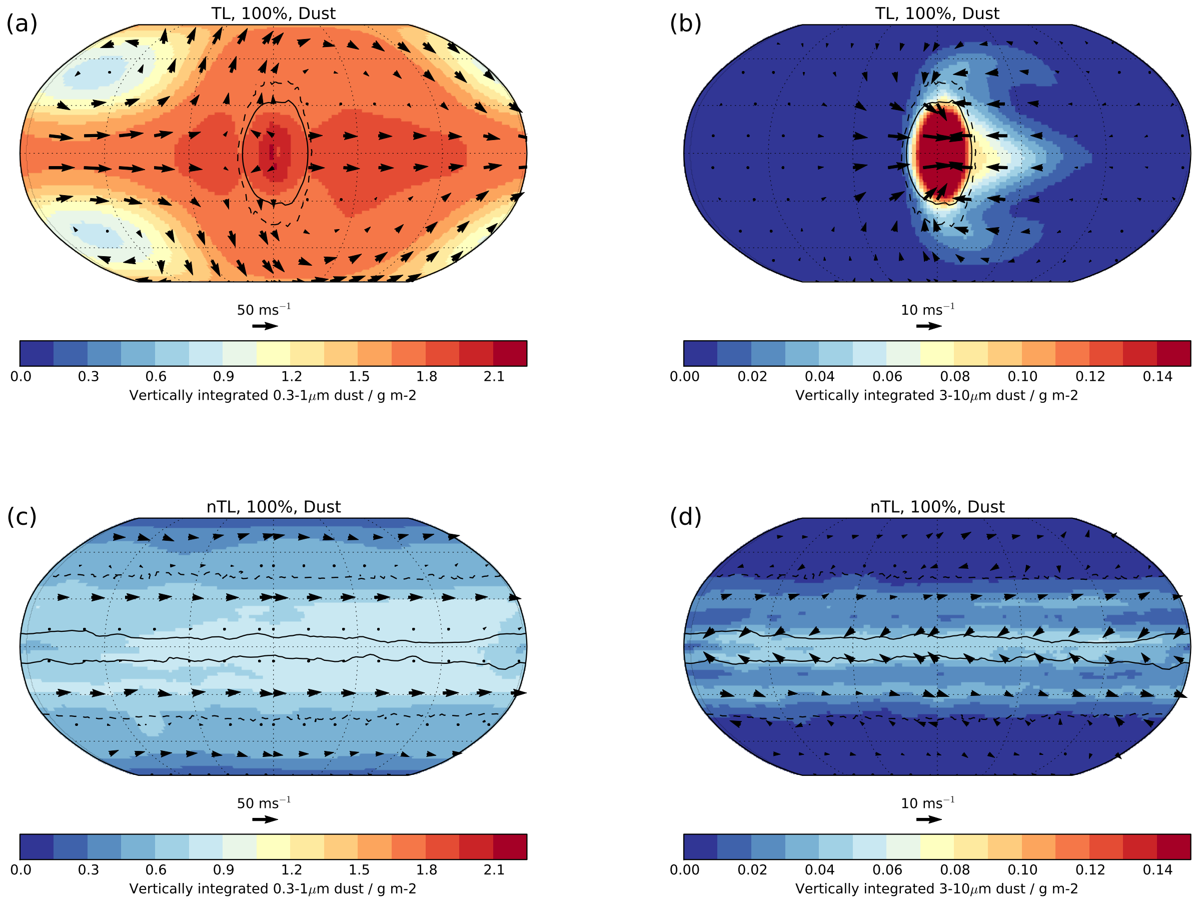

Supplementary Figure 1: Airborne mineral dust distribution. Vertically integrated dust mass mixing ratio from the TL case (a-b) and the nTL case (c-d) with 100% land cover, for small (a,c) and large (b,d) dust sizes. Also shown are the mean (solid) and maximum (dashed) 273 K contours, and wind vectors at 8.5 km ($\approx$ 300 hPa, in a and c) and 10 m (in b and d).

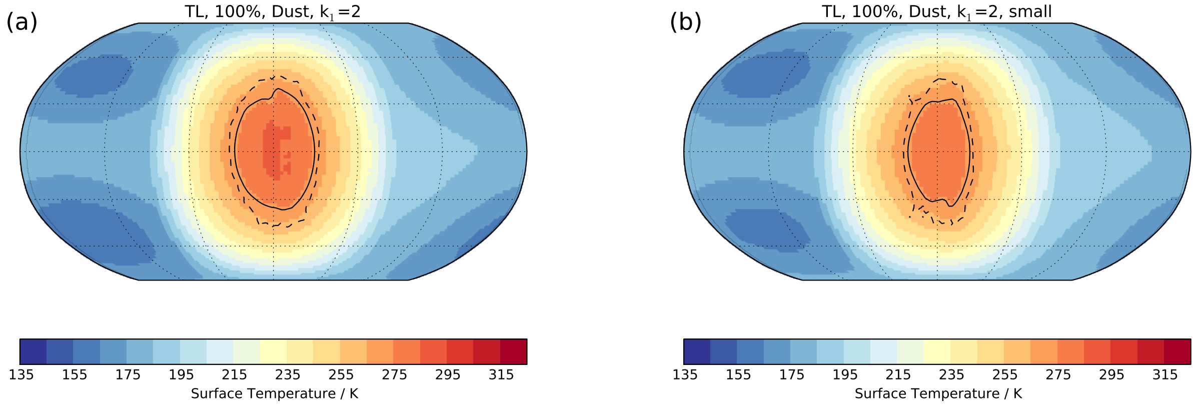

Supplementary Figure 2: Surface temperature sensitivity to dust uplift. Surface temperature from TL experiments with 100% land cover and (a) reduced dust uplift, and (b) reduced uplift plus increased small dust. Also shown are the mean (solid) and maximum (dashed) 273 K contours.

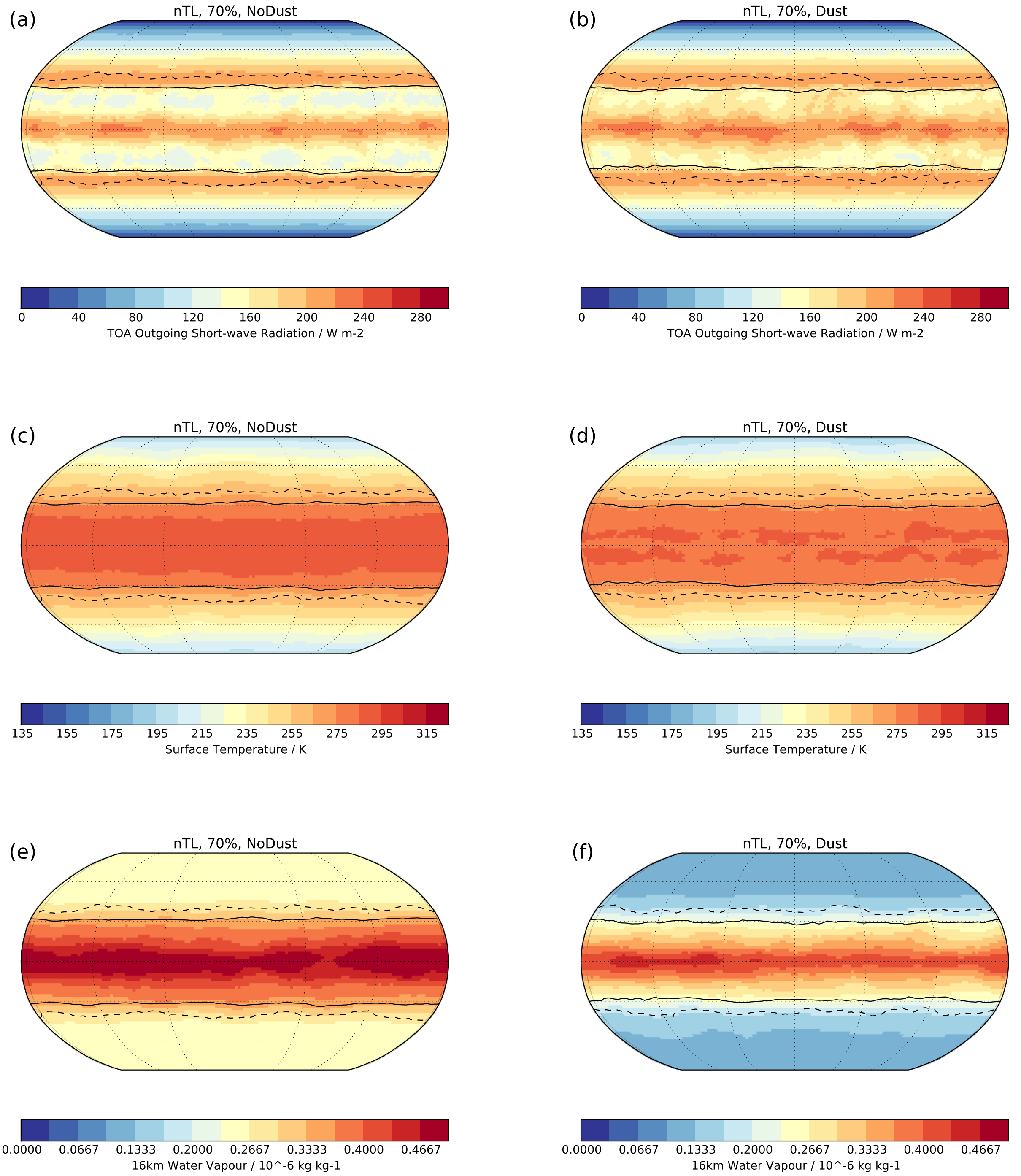

Supplementary Figure 3: Mechanisms on non-tidally-locked planets. Top-of-atmosphere upwelling short-wave radiation (a-b), surface temperature (c-d) and stratospheric water-vapour content (e-f), from the nTL case with 70% land cover for the NoDust (a,c,e) and Dust (b,d,f) simulations. Also shown are the mean (solid) and maximum (dashed) 273 K contours.

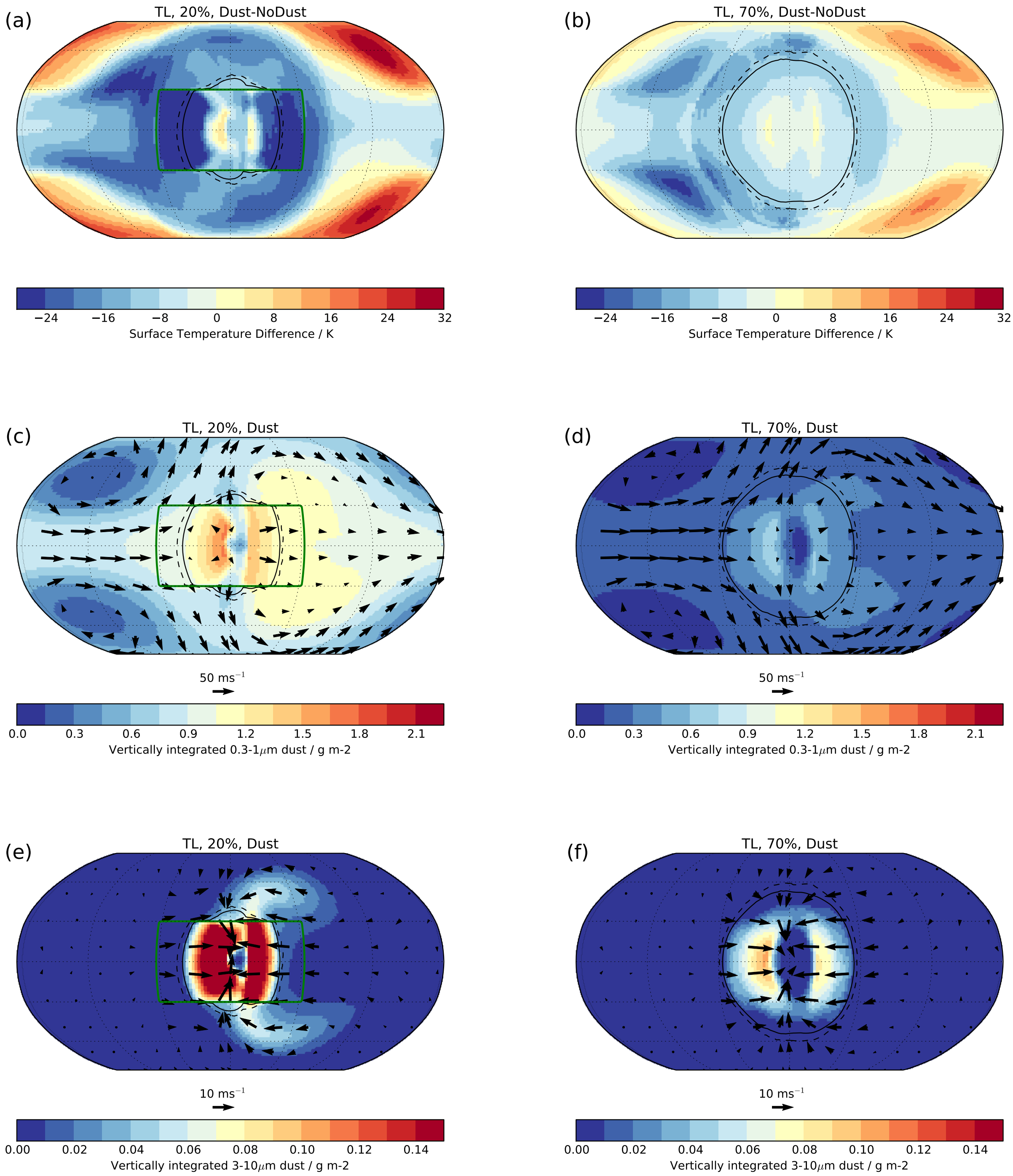

Supplementary Figure 4: Effect of land surface distribution. Difference in surface temperature (Dust - NoDust, a-b), and vertically integrated dust mass mixing ratio (c-f) from the TL case with 20% land cover arranged as a sub-stellar continent (a,c,e, green outline) and 70% land cover arranged globally in each grid-cell (b,d,f). Also shown are the mean (solid) and maximum (dashed) 273 K contours, and wind vectors at 8.5 km ($\approx$ 300 hPa, in c and d) and 10 m (in e and f).

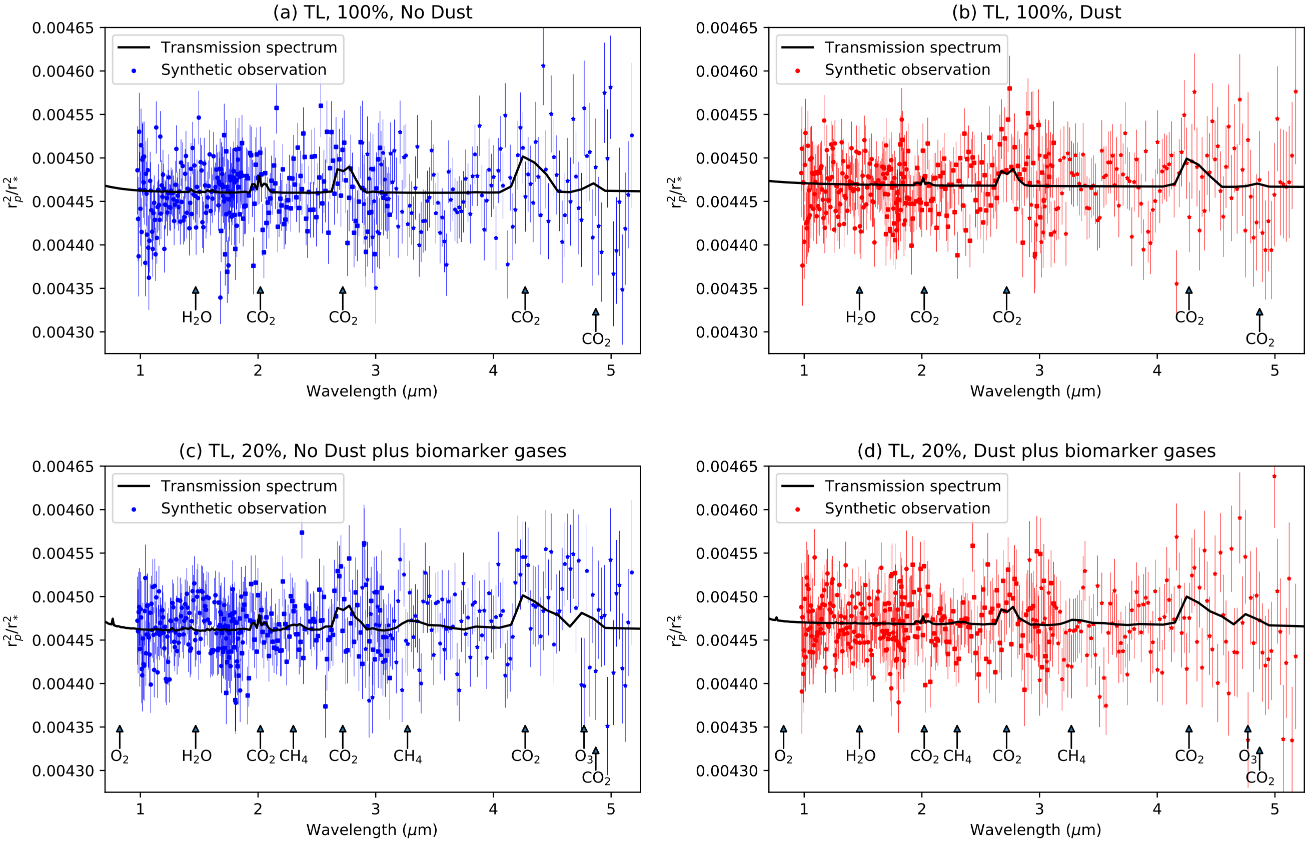

Supplementary Figure 5: Effect of dust on TRAPPIST-1e observations. Simulated transmission spectra (black) and synthetic JWST observations (blue/red), from 15 transits for a dusty (b,d) and non-dusty (a,c) tidally-locked planet, orbiting an M-dwarf of apparent magnitude similar to TRAPPIST-1, with 100% land coverage and no potential biomarker gases (a-b), and 20% land cover and biomarker gases (c-d). A one standard deviation error on the synthetic observation is shown.

Supplementary References

[1] Woodward, S. Modeling the atmospheric life cycle and radiative impact of mineral dust in the Hadley Centre climate model. *J. Geophys. Res.* **106**, 18155–18166 (2001).

[2] Lewis, N. T. *et al.* The influence of a substellar continent on the climate of a tidally locked exoplanet. *The Astrophysical Journal* **854**, 171 (2018).

[3] Boutle, I. A. *et al.* Exploring the climate of Proxima B with the Met Office Unified Model. *Astronomy and Astrophysics* **601**, A120 (2017).

[4] Fauchez, T. J. *et al.* Impact of clouds and hazes on the simulated JWST transmission spectra of habitable zone planets in the TRAPPIST-1 system. *The Astrophysical Journal* **887**, 194 (2019).
